# Production and identification of monoclonal antibodies and development of a sandwich ELISA for detection of the H3-subtype avian influenza virus antigen

**DOI:** 10.1186/s13568-020-00988-7

**Published:** 2020-03-14

**Authors:** Sisi Luo, Xianwen Deng, Zhixun Xie, Jiaoling Huang, Minxiu Zhang, Meng Li, Liji Xie, Dan Li, Qing Fan, Sheng Wang, Tingting Zeng, Yanfang Zhang, Zhiqin Xie

**Affiliations:** Guangxi Key Laboratory of Veterinary Biotechnology, Guangxi Veterinary Research Institute, 51 North Road You Ai, Nanning, 530001 Guangxi China

**Keywords:** H3 subtype, Influenza, Monoclonal antibody, ELISA

## Abstract

The H3 subtype of avian influenza virus (AIV) is widespread in avian species and is frequently isolated in surveillance projects; thus, we have developed a more effective diagnostic approach of a monoclonal antibody (mAb)-based sandwich ELISA for the H3 AIV detection. First, we have produced the essential reagent of mAb against AIV H3 strains with the development of an mAb-Mouse immunization with a purified H3-subtype AIV strain and cell fusion to generate hybridoma cells. These cells were screened with hemagglutination inhibition (HI) tests, and optimal cells were subcloned. We chose a hybridoma cell line that steadily secreted a specific H3-subtype AIV mAb, designated 9F12, that belongs to the IgG1 subclass and has a K-type light chain. 9F12 was shown to bind specifically to the H3-subtype AIV antigen by both immunofluorescence assay and Western blot analysis. Finally, a 9F12-based sandwich ELISA was successfully developed and used to specifically test for this antigen. The sandwich ELISA conditions were optimized, and the specificity and sensitivity were validated. The results for clinical sample detection were consistent with viral isolation. Consequently, the 9F12-based sandwich ELISA is a specific, sensitive, robust, rapid and versatile diagnostic tool for H3-subtype AIV and provides a promising strategy for effective influenza virus prevention and control.

## Introduction

Influenza A virus, a member of the genus *Orthomyxovirus*, is classified into different H and N subtypes according to differences in two major antigen genes, hemagglutinin (HA) and neuraminidase (NA). The primary reservoirs of influenza A virus are waterfowl and shorebirds (Oshansky et al. 2014). The H1–H16 and N1–N9 subtypes have been identified in poultry and wild birds, while H17–H18 and N10–N11 have recently been discovered in bats (Tong et al. 2012, 2013). The H3 influenza A virus is an important subtype that has distinct significance for public health. This subtype has a wide range of hosts, infects mainly avian species and spreads directly to humans across interspecies barriers, circulating in swine, canines, equines, and felines and causing sporadic outbreaks in sea mammals (Bailey et al. 2016). The H3 influenza A virus has different infection characteristics in different hosts. In poultry, H3-subtype avian influenza virus (AIV), the most ubiquitous HA subtype, is frequently isolated from live bird markets (LBMs) in China (Luo et al. 2017). In humans, the pandemic virus A/Hong Kong/1968(H3N2) became endemic after the first year post emergence, and it has since caused yearly seasonal epidemics (Bailey et al. 2016). In swine, the H3N2, H1N1 and H1N2 subtypes are largely responsible for annual outbreaks of swine influenza (Wang et al. 2019). In addition, the avian-origin H3N2 canine influenza virus emerged in dogs in China or Korea in approximately 2005 and has since become an enzootic virus in Southeast Asia and the United States, causing occasional epizootics (He et al. 2019). The H3N8 equine influenza virus was first isolated from horses in Miami, Florida, in 1963 (Kitchen et al. 1963). Currently, it is the only subtype affecting equine populations worldwide and has serious implications for public health (Alves Beuttemmuller et al. 2016; Sreenivasan et al. 2018). In addition, an H3N8 influenza virus carrying mammalian adaptation mutations has been isolated from seals (Solorzano et al. 2015).

The H3 influenza A virus plays unique roles in different hosts. Avian species are primary reservoirs of influenza A virus and can harbor viruses with significant pandemic potential. AIV is a pathogen of economic significance to the poultry industry, and the H3-subtype AIV plays an important role in the emergence of zoonotic infections. Some avian-origin H3N2 viruses transmitted to dogs in South Korea cause acute respiratory disease (Song et al. 2008). H3N2-subtype AIV originating in ducks has been found to acquire the potential to infect humans after multiple infections in a pig population (Shichinohe et al. 2013). Reassortment may occur between H3N2 and other influenza virus subtypes, and an H3N2 isolate containing genes from H7N3 and H7N7 has been found to show the highest sequence homology to the H7N9 virus (Li et al. 2016). H3N2-subtype AIV poses a clear threat to human health, and ongoing surveillance of H3N2-subtype AIV in birds is warranted (Guan et al. 2019); thus, we think it is critical to develop an antibody and diagnostic reagent for H3-subtype AIV. In our study, we produced a monoclonal antibody (mAb) against H3-subtype AIV and developed a sandwich enzyme-linked immunosorbent assay (ELISA) method for the detection of this subtype.

The traditional diagnostic method for H3-subtype AIV involves viral isolation and identification. These techniques are time consuming and tedious, require biosafety facilities and exhibit some limitations in practical application. Currently, detection methods for H3-subtype AIV are urgently needed as technical reserves, and mAbs and sandwich ELISA methods based on mAbs against H3-subtype AIV have rarely been reported. mAbs have a single epitope, display high specificity and have been widely adopted for medical examination. ELISA is highly suitable for epidemiological analyses involving a large number of samples, and sandwich ELISA methods based on mAbs have been developed for other pathogens (Chen et al. 2019; Huang et al. 2019; Zhang et al. 2018). This study had two purposes. First, we sought to produce an mAb against H3-subtype AIV in hybridoma cells and characterize its reactivity. Second, we sought to develop a sandwich ELISA method based on the mAb that could be used as an effective tool for the diagnosis of H3-subtype AIV.

## Materials and methods

### Cells, viruses and sera

The Madin-Darby canine kidney (MDCK) and mouse myeloma (SP2/0) cell lines were purchased from the China Center for Type Culture Collection (CCTCC). The two cell lines were maintained in Dulbecco’s modified Eagle medium (DMEM) (Gibco, USA) supplemented with 10% fetal bovine serum (FBS) (Gibco, USA) and penicillin (100 U/mL)-streptomycin (100 µg/mL). The H3-subtype AIV strains and other viruses used in this study are shown in Table 1. Viral stocks were grown in 10-day-old specific pathogen-free (SPF) embryonated eggs or in MDCK cells, as stated in the text. Polyclonal sera were obtained from H3-positive and H3-negative SPF chickens.

**Table 1:** Origins of the H3-subtype AIV isolates used in this study

Number	Strain name	Source	Whether Hemagglutination was inhibited by the mAb 9F12 using HI test	This sandwich ELISA assay
1	A/Duck/Guangxi/015D2/2009(H3N2)	a	Y	+
2	A/Chicken/Guangxi/015C10/2009(H3N2)	a	Y	+
3	A/Goose/Guangxi/020G/2009(H3N8)	a	Y	+
4	A/Pigeon/Guangxi/020P/2009(H3N6)	a	Y	+
5	A/Duck/Guangxi/057D6/2010(H3N2)	a	Y	+
6	A/Chicken/Guangxi/073C2/2010(H3N2)	a	Y	+
7	A/Duck/Guangxi/112D4/2012(H3N2)	a	Y	+
8	A/Chicken/Guangxi/125C8/2012(H3N2)	a	Y	+
9	A/Pigeon/Guangxi/128P9/2012(H3N2)	a	Y	+
10	A/Chicken/Guangxi/135C10/2013(H3N2)	a	Y	+
11	A/Duck/Guangxi/135D20/2013(H3N2)	a	Y	+
12	A/Goose/Guangxi/139G20/2013(H3N2)	a	Y	+
13	A/Chicken/Guangxi/165C7/2014(H3N2)	a	Y	+
14	A/Duck/Guangxi/175D12/2014(H3N6)	a	Y	+
15	A/Chicken/Guangxi/252C6/2016(H3N2)	a	Y	+
16	A/Duck/Guangxi/272D18/2016(H3N2)	a	Y	+
17	A/Chicken/Guangxi/284C1/2017(H3N2)	a	Y	+
18	A/Pigeon/Guangxi/286P45/2017(H3N2)	a	Y	+
19	A/Pigeon/Guangxi/288P43/2017(H3N6)	a	Y	+
20	A/Goose/Guangxi/318G39/2018(H3N2)	a	Y	+
21	A/Chicken/Guangxi/117B3/2018(H3N2)	a	Y	+
22	A/Duck/Guangxi/030D/2009(H1N1)	a	N	−
23	A/Duck/HK/77/76(H2N3)	b	N	−
24	A/Duck/Guangxi/125D17/2012(H4N2)	a	N	−
25	A/Duck/Guangxi/1/04(H5N1)	a	N	−
26	A/Duck/Guangxi/GXd-5/2010(H6N1)	a	N	−
27	A/Chicken/NY/273874/03(H7N2)	c	N	−
28	A/Turkey/Ontario/6118/68(H8N4)	b	N	−
29	A/Chinese Francolin/Guangxi/020B7/2010(H9N2)	a	N	−
30	A/Duck/HK/876/80(H10N3)	b	N	−
31	A/Duck/PA/2099/12(H11N9)	c	N	−
32	A/Duck/HK/862/80(H12N5)	b	N	−
33	A/Gull/Md/704/77(H13N5)	b	N	−
34	A/Mallard/Astrakhan/263/82(H14N5)	d	N	−
35	A/Shearwater/Western Australia/2576/79(H15N9)	d	N	−
36	A/Shorebird/Delaware/168/06(H16N3)	d	N	−
37	NDV F48	e	N	−
38	ARV S1133	e	U	−
39	EDSV	e	N	−
40	FAdV4-GX001	a	U	−
41	IBV M41	e	U	−

*Y* yes, *N* no, *U* the HI test was unavailable for the strain

a. Guangxi Veterinary Research Institute, China

b. University of Hong Kong, China

c. University of Pennsylvania, USA

d. University of Connecticut, USA

e. China Institute of Veterinary Drug Control, China

### Production of the mAb

The strain A/Duck/Guangxi/112D4/2012(H3N2) was chosen as an immunogen for preparation of the H3 mAb. The impurities were removed via low-speed centrifugation at 4500 rpm for 10 min, and then the viral supernatant was further purified by ultracentrifugation at 32,000 rpm for 90 min. The viral particle pellet was resuspended using phosphate-buffered saline (PBS) at 4 °C overnight and finely purified by sucrose density gradient ultracentrifugation through a 10%/50% sucrose cushion. To inactivate the H3 virus, β-propiolactone was added, and the virus was incubated at 4 °C for 16-24 h. Successful inactivation was confirmed by a lack of growth under chick embryo propagation. Six-week-old female SPF BALB/c mice were inoculated subcutaneously at multiple sites with an emulsion of the inactive H3 virus with an equal amount of Freund’s complete adjuvant. At 21 days and 35 days after the first injection, the second and third immunizations, respectively, were performed with the same antigen and Freund’s incomplete adjuvant. At 49 days after the first injection, the antibody titers against H3-subtype AIV in the mice were detected by hemagglutination inhibition (HI) test. BALB/c mice with high antibody titers were selected, and immunity was boosted by intraperitoneal inoculation of the antigen without adjuvant 3 days before cell fusion.

Spleen cells were harvested from the immunized mice. SP2/0 cells and spleen cells were fused at a ratio of 1:5 with polyethylene glycol (PEG) 4000. The treated cells were suspended in HAT (RPMI 1640 medium containing 20% FBS, 100 mg/mL streptomycin, 100 U/mL penicillin, 100 mM hypoxanthine, 16 mM thymidine, and 400 mM aminopterin) and plated into 96-well tissue culture plates at a density of 1 × 10^5^ cells per well in 200 µL of medium. After cultivation at 37 °C in 5% CO_2_ for 9 days, 12 days, and 15 days, the medium was assayed for H3-subtype AIV-specific antibodies by HI test. Positive hybridoma cells with high antibody titers were subcloned three times. The broad-spectrum activity and specificity of the hybridoma cells were assessed with purified AIV (H1–H16 subtypes), Newcastle disease virus (NDV), and egg drop syndrome virus (EDSV) by HI test. Finally, we chose a high-titer antibody and specific hybridoma cells for subsequent research. The selected hybridoma cells were inoculated into BALB/c mice, and ascetic fluid was purified by saturated ammonium sulfate (SAS) precipitation as described previously (Darcy et al. 2011). The mAb isotypes were determined using a Mouse MonoAb-ID Kit according to the manufacturer’s instructions (Sigma, USA).

### Western blot analysis

To investigate the reactivity of the mAb 9F12 with H3-subtype AIV antigen, Western blot was performed according to the kit manufacturer’s instructions (ProteinSimple Wes, part# PS-T001). In brief, the reagents were first prepared, the H3 virus was diluted with HeLa Lysate Control, and 4 parts of diluted lysate were combined with 1 part of 5× Fluorescent Master Mix in a microcentrifuge tube. The mixture was then heated at 95 °C for 5 min to denature the sample. Second, the sample and reagents were pipetted into the specified plate; the mAb acted as the primary antibody, and a horseradish peroxidase (HRP)-labeled goat anti-mouse antibody (Sigma, USA) acted as the secondary antibody. Finally, Western blot was completed with a fully automated instrument (ProteinSimple Wes, ProteinSimple Corp. 2014).

### Immunofluorescence assay (IFA)

MDCK cells cultured in 48-well plates were inoculated with the strain 112D4(H3) at a multiplicity of infection (MOI) of 0.1 and incubated at 37 °C in 5% CO_2_ for 1 h. In addition, MDCK cells were inoculated with PBS as mock-infected controls. Following infection, viral growth medium consisting of fresh DMEM with 0.3% bovine serum albumin (BSA), 1 µg/ml tolylsulfonyl phenylalanyl chloromethyl ketone (TPCK)-treated trypsin and penicillin (100 U/mL)-streptomycin (100 µg/mL) was added, and the cells were further incubated at 37 °C in 5% CO_2_ for 48 h. The infected cells were fixed with ice-cold methanol for 20 min at room temperature. After washing the cells three times with PBS, blocking buffer was added, and the cells were incubated at 37 °C for 30 min. The cells were then gently washed with PBS, the hybridoma culture supernatant or diluted murine ascetic fluid was added, and the cells were incubated at 37 °C for 1 h. The cells were again washed, and FITC-conjugated goat anti-mouse IgG (Sigma, USA) was added at a dilution of 1:1000. The cells were incubated for 1 h at 37 °C, washed again and observed by fluorescence microscopy.

### Development of a sandwich ELISA for H3-subtype AIV antigen detection

A sandwich ELISA for the H3-subtype AIV antigen was developed using H3-positive polyclonal serum as the coating antibody, the mAb 9F12 as the capture antibody and HRP-conjugated goat anti-mouse IgG as the detection antibody. The basic protocol was composed of the following steps: coating of the ELISA plate with H3 polyclonal serum, three washes with PBS with 0.05% Tween (PBST), addition of blocking buffer with 5% skim milk, incubation, three washes with PBST, addition of the antigen sample, incubation, three washes with PBST, addition of HRP-conjugated goat anti-mouse IgG, incubation, three washes with PBST, addition of 0.24 mg/mL 3,3′,5,5′-tetramethyl benzidine and 0.003% H_2_O_2_ (TMB) substrate, and termination of the reaction with H_2_SO_4_. The working concentrations and incubation times for the polyclonal antibody, antigen, mAb, HRP-conjugated goat anti-mouse IgG, and other components of this ELISA were optimized though the use of checkerboard titrations. The optimal conditions were determined by evaluating the optical density (OD) values at 450 nm (OD_450nm_) and the positive/negative (P/N) ratios of the samples.

### Determination of the cutoff value for the sandwich ELISA

To determine the cutoff value for the sandwich ELISA, 60 H3-free samples, including chicken cloacal swab samples and tissue samples, were tested with the developed sandwich ELISA, and the average (X) and standard deviation (SD) of the OD_450nm_ values of the 60 samples were calculated. X + 3SD was the cutoff value. A sample with an OD_450nm_ greater than or equal to the cutoff value was considered positive, and a sample with a value less than the cutoff value was considered negative.

### Validation of the specificity and sensitivity of the sandwich ELISA

H3 subtype isolates and isolates of other AIV subtypes (H1–H2, H4–H16), NDV, avian reovirus (ARV), EDSV, fowl adenovirus 4 (FAdV4) and infectious bronchitis virus (IBV) (Table 1) were analyzed by sandwich ELISA to evaluate the specificity and cross-reactivity of the method.

To validate the sensitivity of the method, 10, 100, 10^3^, 10^4^, 10^5^, 10^6^, 10^7^, and 10^8^ dilutions of the H3 virus (HA titer 2^8^) were created, and these dilutions were tested with the developed sandwich ELISA.

### Clinical samples

A total of 180 swab samples (oral pharyngeal and cloacal swabs from the same bird were pooled as a single sample) were obtained as part of the AIV surveillance program in LBMs in Nanning, the capital of Guangxi Province, from 2018 to 2019. The swab solutions were tested with the developed H3-subtype AIV sandwich ELISA method. In parallel, 10-day-old SPF embryonated chicken eggs were inoculated with the swab solutions for viral isolation, and allantoic fluid was collected and analyzed by HI test, also amplified with conventional RT-PCR followed by HA and NA amplicon sequencing.

### Statistical analysis

All data are presented as the means with SDs from three independent experiments and were visualized in GraphPad Prism 5.0 software (GraphPad Software, CA, USA).

## Results

### Characterization of the mAb

**Fig. 1:**
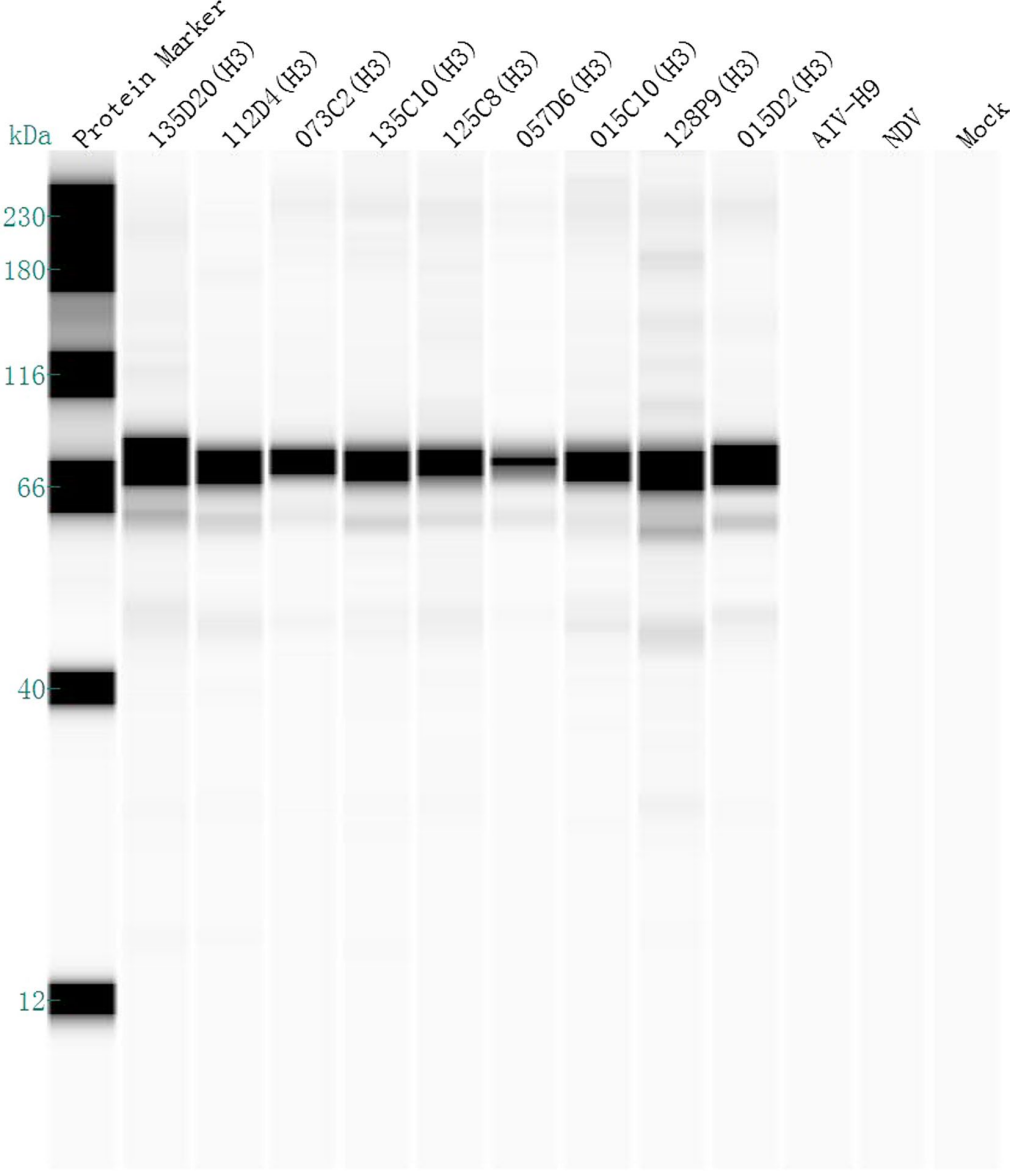
The reactivities of different H3 isolates with the mAb 9F12 were determined in a Western blot assay

**Fig. 2:**
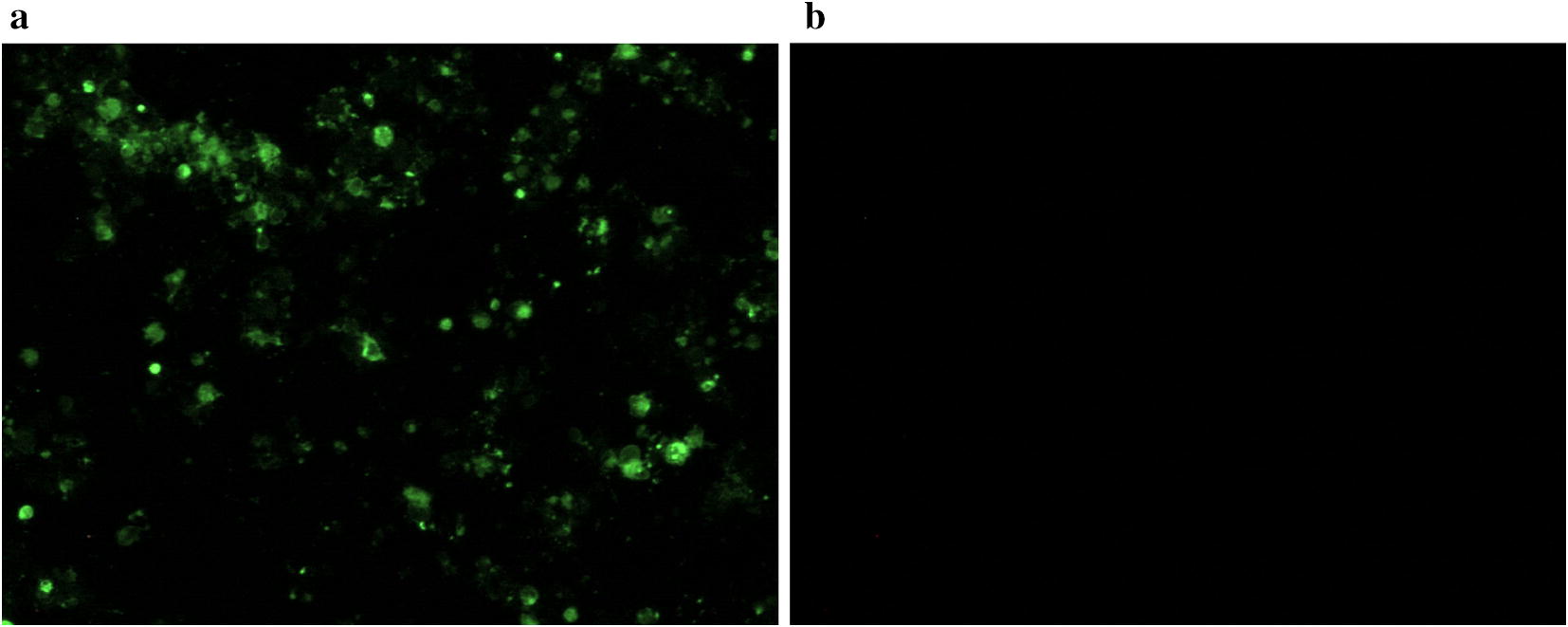
IFA of the mAb 9F12. H3-subtype AIV-infected or mock-infected MDCK cells were fixed with ice-cold methanol and then incubated with the mAb 9F12. After incubation with a FITC-conjugated goat anti-mouse IgG secondary antibody, the cells were visualized by fluorescence microscopy. **a** H3-subtype AIV-infected MDCK cells. **b** Mock-infected MDCK cells

Two hybridoma cells secreted antibodies against H3-subtype AIV that were isolated and screened by the HI test. After three cycles of subcloning, 1 mAb, designated 9F12, had broad-spectrum reactivity to H3-subtype AIV strains and was chosen for subsequent analyses. The mAb 9F12 reacted with the H3 isolates tested but lacked cross-reactivity with other AIV subtypes, NDV and EDSV, as assessed by the HI analysis (Table 1). The hemagglutination of H3-subtype AIV was inhibited by hybridoma supernatants and ascites, and the inhibition test titers were 2^6^–2^8^ and 2^16^–2^18,^ respectively. The heavy-chain subclass of 9F12 was determined to be IgG1, and the light-chain type was K. Nine H3 isolates were randomly chosen for identification of the reactivity of the mAb 9F12 by Western blot assay, and the results suggested that the H3 mAb could react with all nine H3 isolates, which showed specific bands at 66 kDa; in contrast, the AIV-H9, NDV and mock reactions had no band (Fig. 1). IFA using the mAb 9F12 as the primary antibody revealed strong green fluorescence in infected cells (Fig. 2a); mock-infected cells were stained in parallel, but no positive signal was observed (Fig. 2b). The mAb 9F12 was thus used to develop a sandwich ELISA.

### Protocol of the sandwich ELISA for H3-subtype AIV antigen detection

First, 96-well polystyrene plates were precoated with H3-positive polyclonal serum (1:160 dilution) and incubated at 37 °C for 1 h and at 4 °C overnight. The plates were then blocked with 5% skim milk at 37 °C for 1 h. Antigen samples were distributed into each well, and the plates were incubated at 37 °C for 1 h. The mAb 9F12 was then diluted 1:100 in 1% BSA and added to the plates, which were again incubated at 37 °C for 1 h. HRP-conjugated goat anti-mouse IgG was diluted to 1:1000 with 1% BSA and added to the plates before incubation at 37 °C for 1 h. After each incubation and before adding a new reagent, the plate wells were washed three times using PBST to remove unbound reagent. The plates were thoroughly washed, and 100 μL of a TMB substrate solution was added. Following incubation at room temperature in the dark for 10 min, the chromogenic reaction was quenched with 50 μL of 0.5 M H_2_SO_4_, and the OD_450nm_ values were measured using an ELISA plate reader.

### Cutoff value for the sandwich ELISA

To determine the cutoff value for the sandwich ELISA, 60 H3-free samples, including chicken cloacal swab samples and tissue samples, were tested. The average (X) OD_450nm_ was 0.1297, the SD was 0.0415, and the X + 3SD was 0.2542. For an OD_450nm_ ≥ 0.2542, the sample was determined to be positive; for an OD_450nm_ < 0.2542, the sample was determined to be negative.

### Specificity and sensitivity of the sandwich ELISA

**Fig. 3:**
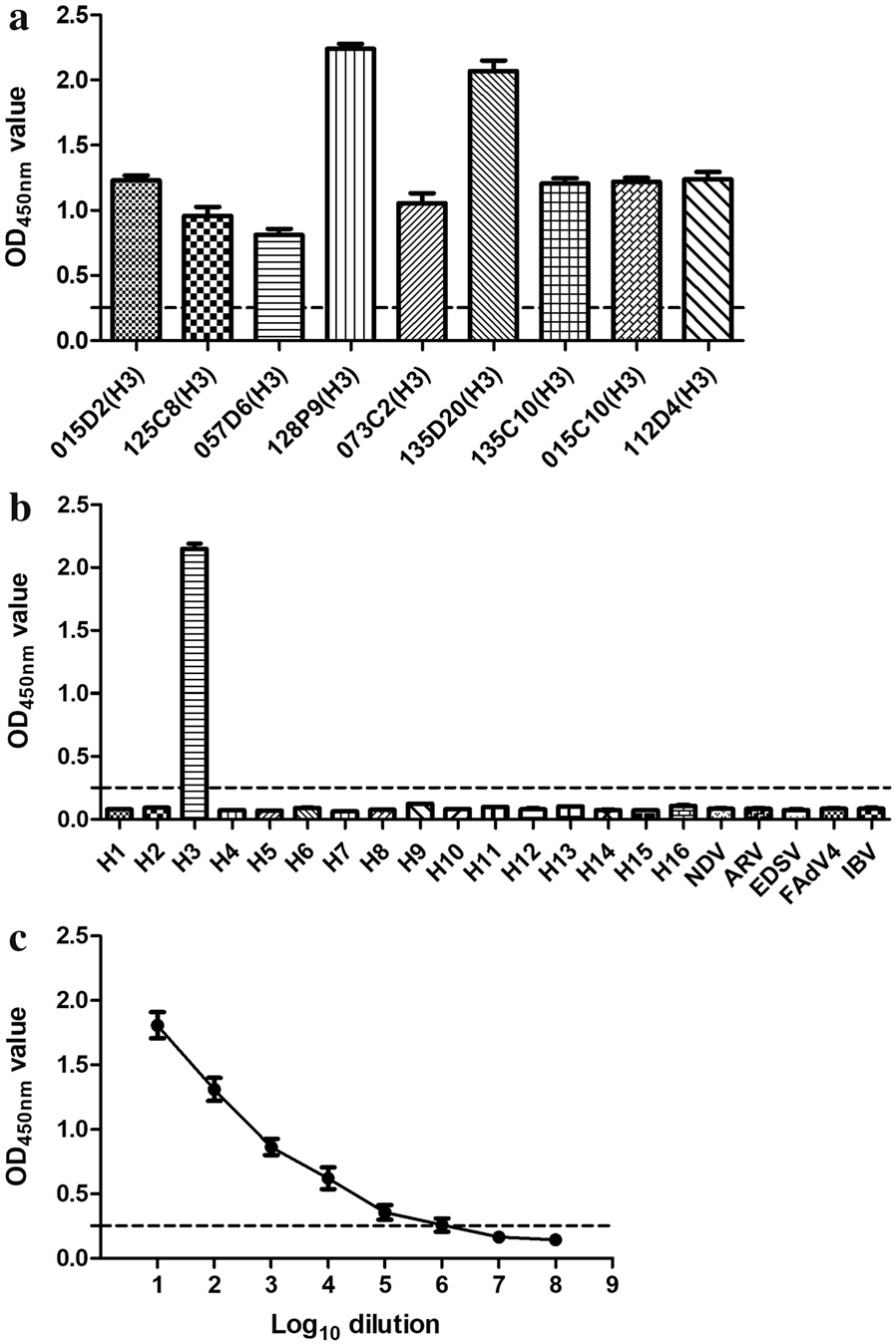
Specificity and sensitivity of the sandwich ELISA. **a** Different H3 isolates were tested and shown to be positive. **b** Other HA subtypes of AIV, NDV, ARV, EDSV, FAdV4 and IBV were tested and shown to be negative. **c** Sensitivity of the sandwich ELISA for H3 virus detection. Dotted line indicates the cutoff value

H3 isolates were subjected to this ELISA, and the results were all positive (Table 1 and Fig. 3a). H1–H2, H4–H16, NDV, ARV, EDS, FAdV4 and IBV were also assayed by the sandwich ELISA, and the OD_450nm_ values were all less than the cutoff value; thus, the samples were considered negative (Table 1 and Fig. 3b). Each sample was assayed three times under the same conditions on different days, and the replicate analyses produced very similar results.

H3-subtype AIV (HA: 2^8^) was diluted 1:10–1:10^5^, and testing revealed that the OD_450nm_ value was greater than the cutoff value. When the H3 virus was diluted 1:10^6^, the OD_450nm_ value fluctuated around the cutoff value. When the dilution was 1:10^7^, the OD_450nm_ value was less than the cutoff value, thus the sample was reported as negative (Fig. 3c). Each sample was assayed three times under the same conditions on different days, and the replicate analyses produced very similar results.

### Detection in clinical samples

Among 180 clinical samples, twenty-three samples were determined to be positive for H3-subtype AIV by the developed sandwich ELISA method. The same 23 samples were also determined to be positive by viral isolation, HI testing and amplicon sequencing (Table 2). The remaining samples were all negative. Therefore, the results of the two methods were coincident.

**Table 2:** Results obtained from clinical samples using this sandwich ELISA and isolation and identification

Host	Sandwich ELISA	Isolation and identification
Duck	H3(12)	H3N2(10), H3N6(1), H3N8(1)
Chicken	H3(7)	H3N2(7)
Goose	H3(3)	H3N2(2), H3N6(1)
Pigeon	H3(1)	H3N2(1)
Total	H3(23)	H3N2(20), H3N6(2), H3N8(1)

## Discussion

H3-subtype AIV is a major subtype threatening both human and animal health. It is important to enhance surveillance for H3-subtype AIV, and a simple, rapid, specific, sensitive and effective detection method suitable for mass detection is urgently needed. Some molecular methods for the detection of H3-subtype AIV have been developed. Thus far, several reports have described the diagnosis of H3-subtype AIV using RT-LAMP, real-time RT-PCR and multiplex RT-PCR (Peng et al. 2011; Tang et al. 2012; Teng et al. 2015). However, serological detection methods for H3-subtype AIV have rarely been reported. Routine monitoring of H3-subtype AIV frequently involves large numbers of samples; notably, the developed ELISA method, which is performed with a 96-well plate, can be used to simultaneously analyze 94 samples in addition to the positive and negative control samples.

In recent years, the use of gene expression or virus-like particles to produce antigens by genetic recombination has yielded antigens with increased purity and concentrations and has enabled long-lasting humoral immunity and cross-protection to be achieved. In our study, direct inoculation of mice with inactive virus kept the epitopes intact and preserved the natural spatial structure of the antigen, thus yielding an antigen that may be superior to those produced by gene expression. It is very likely that a protein does not fully recover its original stereoscopic conformation during the process of protein renaturation. Thus, we attempted to use inactive whole virus to prepare and produce the H3 mAb. Whole viruses have widely been used as immunogens for the production of mAbs to develop ELISAs for the diagnosis of disease (Chen et al. 2019; Huang et al. 2019; Zhang et al. 2018). The HA glycoprotein, the major surface protein of influenza A virus, plays a critical role in viral infection and is a primary target of neutralizing antibodies (Ito et al. 2019). In our study, the mAb was screened by HI assay; thus, the mAb 9F12 was determined to recognize the HA of H3-subtype AIV. The Western blot and IFA results revealed that the mAb 9F12 specifically bound to the H3 virus with effective reactivity. In an analysis of 180 clinical samples, the detection results of this sandwich ELISA were consistent with those of gold standard methods for isolation and identification, but further analysis of more clinical samples is needed to validate this method and extend its use to the routine diagnostic epidemiological detection of H3-subtype AIV infection.

BALB/c mice were inoculated with an inactive local epidemic strain of H3-subtype AIV, and hybridoma cells that could steadily secrete a high titer of antibody specific for H3-subtype AIV was obtained through a series of cell fusion screening, HI testing and subcloning steps. The mAb obtained from the resulting hybridoma cells had good specificity and sensitivity. The successful production of this mAb raised three key points. (1) For immunized mice, a higher antibody titer is associated with a higher success rate. If the antibody titer is low, the levels of corresponding specific antibodies in spleen cells are reduced, leading to a lack of a specific and high-titer mAb in the process of cell fusion. (2) PEG was used for cell fusion and was added within 1–2 min. In addition, attention was paid to the formation of particles. Once particles appear, the remaining PEG must be quickly added. The time of PEG addition must be controlled to avoid excessive fusion. (3) Myeloma cell activity must be ensured because cell morphology is not fully intact, and poor refraction will affect cell fusion, resulting in cell death. In this study, the preparation of the mAb laid a foundation for the establishment of a sandwich ELISA for the detection of H3-subtype AIV.

The sandwich ELISA included mainly the following five components: a polyclonal antibody, a sample, an mAb, a conjugated HRP antibody, and a substrate. The ELISA plate was coated with polyclonal antibody serum, and the antigen sample was added. If the sample had H3-subtype AIV, it combined with the polyclonal antibody to form an antigen–antibody complex. After this step, the wells were rinsed with PBST to remove unbound components. The H3 mAb was then added and reacted with the antigen in the complex, binding to the ELISA plate. An HRP-labeled goat anti-mouse antibody was added to interact with the mAb. The results were determined qualitatively by a color change and quantitatively by the reaction of the TMB substrate, as measured by an ELISA reader. This ELISA allowed samples to first react with the polyclonal antibody and then react with the mAb. The mAb recognized a single antigen epitope. The specificity of the mAb was better than that of the polyclonal antibody, but the binding range was not as wide as that of the polyclonal antibody. The initial combination of clinical samples with polyclonal antibodies for preliminary screening and the subsequent reaction of the samples with the mAb increased the detection rate and prevented false negatives.

The hybridoma cells producing H3-subtype AIV prepared in this study can steadily secrete homogeneous and high-titer antibodies and provide a very useful mAb for further research on H3-subtype AIV. In addition, the sandwich ELISA method is time saving, convenient, highly specific, sensitive and repeatable. Furthermore, the method can produce results without a specific instrument or the need for professional technical staff; it is performed according to a simple protocol and provides an effective method for the diagnosis of H3-subtype AIV.

## Data Availability

All data obtained have been included in the manuscript.
